# Simplified Models of Non-Invasive Fractional Flow Reserve Based on CT Images

**DOI:** 10.1371/journal.pone.0153070

**Published:** 2016-05-17

**Authors:** Jun-Mei Zhang, Liang Zhong, Tong Luo, Aileen Mae Lomarda, Yunlong Huo, Jonathan Yap, Soo Teik Lim, Ru San Tan, Aaron Sung Lung Wong, Jack Wei Chieh Tan, Khung Keong Yeo, Jiang Ming Fam, Felix Yung Jih Keng, Min Wan, Boyang Su, Xiaodan Zhao, John Carson Allen, Ghassan S. Kassab, Terrance Siang Jin Chua, Swee Yaw Tan

**Affiliations:** 1 National Heart Center Singapore, 5 Hospital Drive, Singapore 169609, Singapore; 2 Duke-NUS Medical School, 8 College Rd, Singapore 169857, Singapore; 3 California Medical Innovations Institute, San Diego, CA 92121, United States of America; 4 Department of Mechanics and Engineering Science, College of Engineering, Peking University, Beijing, 100871, China; 5 School of Information Engineering, Nanchang University, Nanchang, Jiangxi, 330031, China; Magna Graecia University, ITALY

## Abstract

Invasive fractional flow reserve (FFR) is the gold standard to assess the functional coronary stenosis. The non-invasive assessment of diameter stenosis (DS) using coronary computed tomography angiography (CTA) has high false positive rate in contrast to FFR. Combining CTA with computational fluid dynamics (CFD), recent studies have shown promising predictions of FFR_CT_ for superior assessment of lesion severity over CTA alone. The CFD models tend to be computationally expensive, however, and require several hours for completing analysis. Here, we introduce simplified models to predict noninvasive FFR at substantially less computational time. In this retrospective pilot study, 21 patients received coronary CTA. Subsequently a total of 32 vessels underwent invasive FFR measurement. For each vessel, FFR based on steady-state and analytical models (FFR_SS_ and FFR_AM_, respectively) were calculated non-invasively based on CTA and compared with FFR. The accuracy, sensitivity, specificity, positive predictive value and negative predictive value were 90.6% (87.5%), 80.0% (80.0%), 95.5% (90.9%), 88.9% (80.0%) and 91.3% (90.9%) respectively for FFR_SS_ (and FFR_AM_) on a per-vessel basis, and were 75.0%, 50.0%, 86.4%, 62.5% and 79.2% respectively for DS. The area under the receiver operating characteristic curve (AUC) was 0.963, 0.954 and 0.741 for FFR_SS,_ FFR_AM_ and DS respectively, on a per-patient level. The results suggest that the CTA-derived FFR_SS_ performed well in contrast to invasive FFR and they had better diagnostic performance than DS from CTA in the identification of functionally significant lesions. In contrast to FFR_CT_, FFR_SS_ requires much less computational time.

## Introduction

Coronary artery disease (CAD) is a very prevalent cardiovascular disease which can lead to angina and myocardial infarction (MI) [1–3]. The quantification of functional coronary stenosis is of high significance for patient management to prevent mortality from CAD [4].

Both anatomical parameters and hemodynamic indices are commonly applied to quantify the severity of CAD. The anatomical parameters of diameter stenosis (DS) and area stenosis (AS) express the diameter and area of a stenosed region, respectively, relative to a “normal” segment. Although computed tomography angiography (CTA) has proven valuable to characterize the anatomic severity of CAD with lower cost and fewer complications, it cannot determine the hemodynamic significance of a stenosis and it has high false positive rate in contrast to a hemodynamic index, such as fractional flow reserve, FFR [5]. FFR is defined as the ratio of maximal blood flow achievable in a stenotic artery to the theoretical maximal flow in the same vessel when stenosis is absent [6]. Assuming a linear pressure-flow relation, flow is proportional to pressure when resistance is constant. Therefore, FFR can be calculated as the ratio of the pressure distal to a coronary stenosis to aortic pressure at the hyperemia state [7]. Because FFR can identify the functionally significant coronary stenoses, including intermediate coronary stenoses [8–10], it is used as gold standard to identify those stenoses that can most likely benefit from percutaneous coronary intervention (PCI). Revascularization is commonly recommended when the coronary stenosis leads to FFR ≤ 0.80. FFR can only be measured via invasive coronary catheterization at hyperemic state, however, which carries higher medical cost and some complications [11]. There have been some alternative adoptions of FFR by either removing the need for adenosine [12] or pressure wire [13] but still require invasive angiography.

Recently, Computational Fluid Dynamics (CFD) has been applied to simulate blood flow to compute FFR_CT_ for patient-specific coronary artery models reconstructed from CTA, with lumped parameter heart and coronary models [14,15]. The multicenter clinical trials of DISCOVER-FLOW, DeFACTO and NXT [16–20] demonstrated that FFR_CT_, derived non-invasively through combining CT images and CFD simulations, improved diagnostic accuracy and discrimination than CT alone in differentiating ischemic and non-ischemic stenoses. The computational time for transient CFD simulation, however, was significant (6 hours[16] or 1–4 hours [19] for CFD analysis per examination), which may limit its utility in the clinic. By modeling vessels as 1D segments in CFD simulation, the computational time was significantly reduced to 5–10 minutes per patient [21]. The latter approach, however, only had moderate to good correlation (Pearson correlation coefficient = 0.59) as compared with invasive FFR.

Since the calculation of FFR is based on time-averaged pressure measured over several cardiac cycles during coronary angiography [12], we hypothesize that non-invasive FFR_SS_ can be obtained from steady state flow simulation using novel boundary conditions while maintaining acceptable accuracy relative to FFR. In this way, the computational time can be reduced to 1/16 of the transit state model as reported in our previous study [22].

An alternative approach to CFD that yields real-time calculation is the use of analytical models. Huo et al. [23] recently proposed an analytical model to allow real-time computation of FFR_AM_. The calculation of FFR_AM_ was based on stenosis dimensions (proximal, distal and minimal lumen area, and length) and hyperemic coronary flow rate through the lesion (i.e., no ad hoc or empirical parameters). This method has been validated in-vitro and in swine coronary arteries [23], but not clinically.

The aim of this pilot study was to compute FFR_SS_ and FFR_AM_ from CTA using steady state flow simulation and analytical model, respectively. The diagnostic performance of FFR_SS_ and FFR_AM_ was validated against the reference standard of invasive FFR.

## Methods

### Study Design

The pilot study was approved by the local institutional ethics review board (SingHealth CIRB 2014/363/C) and conducted in National Heart Centre Singapore. The informed consent was waived, as it is a retrospective study. Patient records/information was anonymized and de-identified prior to analysis. For inclusion in this retrospective study, coronary CTA must have been performed before coronary angiography due to clinical needs. Patients, who had undergone previous coronary interventions or coronary bypass surgery in the vessels of interest, and patients who had a cardiac event and/or PCI in between coronary CTA and invasive angiography were excluded from this study. Patients, who had Agatston coronary artery calcium score greater than 2,000 or non-diagnostic CTA image quality, were also excluded. From March 2010 to April 2013, 35 patients underwent CTA followed by invasive angiography with FFR. A total of 9 patients were excluded, 3 because of undergoing PCI in between CTA and invasive angiography, 5 because of Agatston coronary artery calcium score greater than 2,000, and 1 because of non-diagnostic CTA image quality. In total, 21 patients were recruited and their baseline characteristics were tabulated in Table 1. For the recruited patients, the time-lag between CTA and coronary angiography varied from 3 to 329 days with average time-lag of 48 days. 81% of the recruited patients underwent CTA and coronary angiography within 50 days. The researchers were blinded to invasive FFR values before calculating the FFR_SS_ and FFR_AM_.

**Table 1:** Baseline characteristics.

Variable (n = 21)	Mean ± SD or Frequency (%)
Age (years)	57 ± 10
Male	16 (76%)
Chinese	18 (86%)
Hypercholesterolemia	13 (62%)
Hypertension	11 (52%)
Diabetes	2 (10%)
Current smoker	2 (10%)
Previous percutaneous coronary intervention^+^	1 (5%)
**Symptoms**	
Chest Pain	9 (43%)
Shortness of Breath	7 (33%)
**Laboratory measures**	
Hemoglobin (g/dl)	13.6 ± 1.6
Hematocrit (%)	40.7 ± 4.3
Creatinine (μmol/L)	82.8 ± 15.8
Body-mass index (kg/m^2^)	24.9±4.5
**Medications**	
Aspirin	13 (62%)
Beta-blocker	7 (33%)
Nitrate	5 (24%)
Statins	15 (71%)
ACE inhibitors	1 (5%)
Calcium-channel blockers	4 (19%)
Clopidogrel	4 (19%)
ARBs	5 (24%)

Notes: ACE represents angiotensin-converting enzyme, ARB represents angiotensin receptor blocker

^+^ Not in the vessel territories interrogated with invasive FFR measurement.

### Coronary CTA Acquisition

Standard coronary CTA was performed by using a 64 slice multi-detector CTA scanner (Toshiba Aquilion 64, 0.5mm × 64 detector row) in 1 (5%) patient (for patient #6 of Table 2) and a 320 slice multi-detector CTA scanner (Toshiba Aquilion ONE^TM^, 0.5mm × 320 detector row) in the rest of 20 patients following current clinical guidelines [24]. The scanning range was planned individually to cover the major coronary arteries and aorta. Beta-blocker was administrated in 4 (19%) patients to moderate heart rate. Omnipaque non-Ionic contrast medium was routinely given before image acquisition to highlight regions of interest in the CT images. The dose of contrast was controlled by using electrocardiographic triggering prospectively. Image acquisition was performed with an inspiratory breath-hold. The scan parameters were gantry rotation time at 350–400 ms, tube potential at 100–120 kV and field of view (FOV) at 161–230mm. Depending on the cardiac dimensions and pitch, scanning time was 5.7–8.4 s for a single breath hold in the craniocaudal direction. All CT images were recorded with 0.25 mm increment (i.e., 0.5 mm slice thickness) and saved in 'DICOM' format' for image processing to reconstruct 3D patient-specific coronary models. The mean total Agatston coronary artery calcium score was 333±370 for the recruited patients.

**Table 2:** Invasive and non-invasive hemodynamic indices of patients with CAD.

Patient No	Location of Stenosis	FFR	FFR_SS_	FFR_AM_
1	LAD Mid	0.74	0.73	0.67
2	LAD Mid	0.83	0.87	0.93
3	LAD Mid	0.94	0.93	0.92
3	LAD D1	0.86	0.86	0.89
4	LAD Proximal	0.97	0.98	0.97
5	LAD Ramus	0.89	0.88	0.88
5	LAD D1	0.93	0.81	0.84
6	LAD Mid	0.78	0.78	0.69
7	R-PLB	0.71	0.72	0.85
8	RCA Distal	0.91	0.81	0.84
9	LAD Distal	0.73	0.73	0.64
9	LCX Mid	0.92	0.87	0.89
9	LCX OM1	0.95	0.84	0.95
10	LAD Mid	0.85	0.82	0.95
10	LAD D1	0.79	0.82	0.72
11	LAD Proximal	0.78	0.69	0.74
12	LAD Mid	0.83	0.88	0.88
13	LAD Mid	0.89	0.85	0.84
14	LAD Proximal	0.91	0.88	0.89
15	LAD Mid	0.85	0.75	0.78
16	RCA Mid	0.98	0.94	0.96
16	LAD Mid	0.81	0.81	0.79
16	LAD D2	0.63	0.67	0.51
17	RCA Proximal	0.95	0.85	0.87
18	LAD Mid	0.93	0.90	0.92
18	D1	0.92	0.92	0.91
18	RCA Mid	0.93	0.89	0.88
19	LAD Proximal	0.76	0.73	0.83
20	LAD Mid	0.86	0.87	0.88
20	LAD D1	0.84	0.84	0.94
21	LAD Mid	0.74	0.82	0.75
21	LAD Distal	0.66	0.67	0.47

Notes: LAD: left anterior descending artery; LCX: left circumflex artery

D1: first diagonal branch; R-PLB: right posterior descending artery

RCA: right coronary artery; OM1: first obtuse marginal branch.

### Coronary Angiography and FFR Measurement

Coronary angiography was performed via percutaneous femoral approach according to standard guidelines [25]. For the recruited patients, invasive FFR measurement was performed due to clinical needs, according to the institutional protocol. Omnipaque non-Ionic contrast medium was routinely given during angiographic procedure to highlight the regions of interest. Pressure measurement was conducted in at least 1 vessel with pressure-sensor-tipped wire (RADI, Volcano or St Jude Medical pressure wire), followed by intravenous infusion (for patient #19 of Table 2 at dose of 60 μg/kg/min) or intracoronary bolus (for the rest 20 patients at dose of 60 to 200μg) of vasoactive substances (adenosine) to create maximal hyperemia. More patients underwent FFR measurements followed by intracoronary bolus injection of adenosine because of its advantages like shorter duration and better patient comfort. It has been reported, however, that intravenous infusion of adenosine yielded identical FFR result compared with intravenous infusion [26]. During maximum hyperemia, pressure measurements were repeated to calculate FFR. FFR ≤ 0.8 indicated a hemodynamically significant coronary stenosis.

### Image Processing for Reconstructing Patient Specific Model

Image processing was performed to segment the artery structure from the background CT image for reconstructing the 3D coronary artery tree. At first, a Hessian matrix-based filter was applied to each single transverse CT image to obtain high order geometric characteristics; i.e., the principal curvature of image intensity [27]. Eigenvector analysis of the Hessian matrix obtained was used to determine whether the voxel under analysis belonged to the vessel structure or not. Finally, the graph-cuts based image segmentation technique [28] was applied to the enhanced 3D image, before using marching cube algorithm to establish the triangulated surface mesh from segmented voxel data. Manual editing was necessary for some models to remove artifacts and small vessels with diameters < 1mm. This image processing algorithm has been previously validated [29].

Both AS and DS were calculated based on the reconstructed coronary artery tree model. AS (DS) was defined as 100% minus the percentage of minimal lumen area (diameter) to the reference lumen area (diameter) at the most appropriate reference site. The reference site was comprised of a non-diseased arterial segment in close proximity to the lesion, preferably with no intervening branch vessels [30]. As recommended in SCCT guideline [24], coronary stenoses can be graded as minimal (DS<25%), mild (25%≤DS≤49%), moderate (50%≤DS≤69%), severe (70%≤DS≤99%) and occlusion (DS = 100%) stenoses separately. Following the guideline, a stenosis with DS ≥ 50% was considered as an anatomically significant stenosis. Although AS was believed to be more accurate and less dependent on the projection plane and reference site than DS [30], there are only a few studies on its reproducibility and accuracy. As suggested by a recent study [31], AS ≥ 62% was used as a threshold to identify the ischemic coronary lesions.

### CFD Simulation to Derive FFR_SS_

ANSYS workbench, a commercial software, was used to discretize the computational domain into tetrahedral shaped elements. After mesh dependency test, the coronary artery tree model was discretized with a total of about 0.8 million volume cells. Further grid refinement led to < 1% relative error in maximum velocity.

FLUENT^TM^ was used to solve continuity and Navier-Stokes equations. Blood was modeled as Newtonian fluid to simulate blood flow in the patient-specific coronary artery tree models. Appropriate boundary conditions were specified at the inlet and outlets of each model respectively to mimic physiological conditions (see details in S1 Appendix), and satisfy 3 key principles, which were also applied in [16,19]. First, coronary supply was assumed to meet myocardial demand at rest. Hence, resting total coronary blood flow rate can be calculated from myocardial mass which were assessed from CT images [32,33]. Second, the resistance of each coronary branch at rest was proportional to the size of parent and child branch vessels [34]. Third, coronary resistance was assumed to reduce predictably during hyperemia conditions (see details in S1 Appendix), which was within the physiological range measured by Wilson et al. [35].

In contrast to previous studies [16,19], steady flow simulation with a novel iterative boundary condition was employed in this study, which reduced the computational cost significantly in contrast to using pulsatile flow simulation. All computations were executed in a Dell T7500 workstation and it took 0.5 to 2 hours of computational time for one case. The computational time could be further reduced with more advanced hardware.

### Analytical Calculation to Obtain FFR_AM_

Huo et al. [23] proposed an analytical model to predict FFR based on stenosis dimensions and hyperemic coronary flow with no empirical parameters, which has been validated in-vitro and in vivo in swine. We used this model for calculating FFR_AM_ for human data. The analytical model was derived from energy conservation which considered the convection and diffusive energy losses as well as the energy loss due to sudden constriction and expansion in lumen area. In this study, the hyperemic coronary flow rate through the coronary branches was specified with the data obtained from simulation. Together with the dimensions (proximal, distal and minimal lumen area, and length) of the stenosis, the pressure drop over a stenosis (∆P_1_) was derived according to the analytical model (see S2 Appendix). The pressure drop over the normal vessel segments (from the inlet to the segment before stenosis), ∆P_2_, was calculated according to Poiseuille equation. Accordingly FFR_AM_ was calculated by 1-(∆P_1_+∆P_2_)/*P* where *P* represented patient-specific mean aortic pressure. In this way, FFR_AM_ was computed real-time.

### Statistical Analysis

To assess the diagnostic performance of FFR_SS_ and FFR_AM_, the clinical standard (FFR ≤ 0.8) was used as a reference. A patient was considered positive if any vessel had FFR ≤ 0.8. The same threshold (0.8) was applied for both FFR_SS_ and FFR_AM_. To compare with the diagnostic performance of CTA, DS ≥ 50% [30] and AS ≥ 62% [31] were used as the threshold to identify the ischemic lesions in this study. For statistical analysis, all continuous variables were reported as means ± standard deviations and absolute variables were presented as totals or percentages. Pearson correlation coefficient, Bland-Altman analysis and the area under the receiver-operating characteristic curve (AUCs) were calculated to determine the correlations, differences and diagnostic performance of variable indicators, respectively. All statistical analyses were performed using SPSS version 16.0 and MedCalc version 16.2.1.

## Results

### Patient Characteristics

Table 1 shows the baseline characteristics of the study cohort. As a pilot study, 21 patients (aged 57±10 years, 16 males and 5 females) underwent CTA and invasive angiography tests; 43% of patients had chest pain, while 33% had shortness of breath. There were no adverse events or revascularization between the tests. FFR was invasively measured in a total of 32 coronary vessels (as shown in Table 2) where 84% (n = 27) of the stenosed vessels were located in the left coronary artery tree. Of the 32 vessels, the invasively measured FFR was 0.85±0.09 and 31% (n = 10) of the vessels had functionally significant stenoses (FFR≤0.8). The SYNTAX score [36] was 2 to 34.5 for the recruited patients with a mean value of 11.3.

### Assessment of Functional Significance of Coronary Artery Stenosis

**Fig 1:**
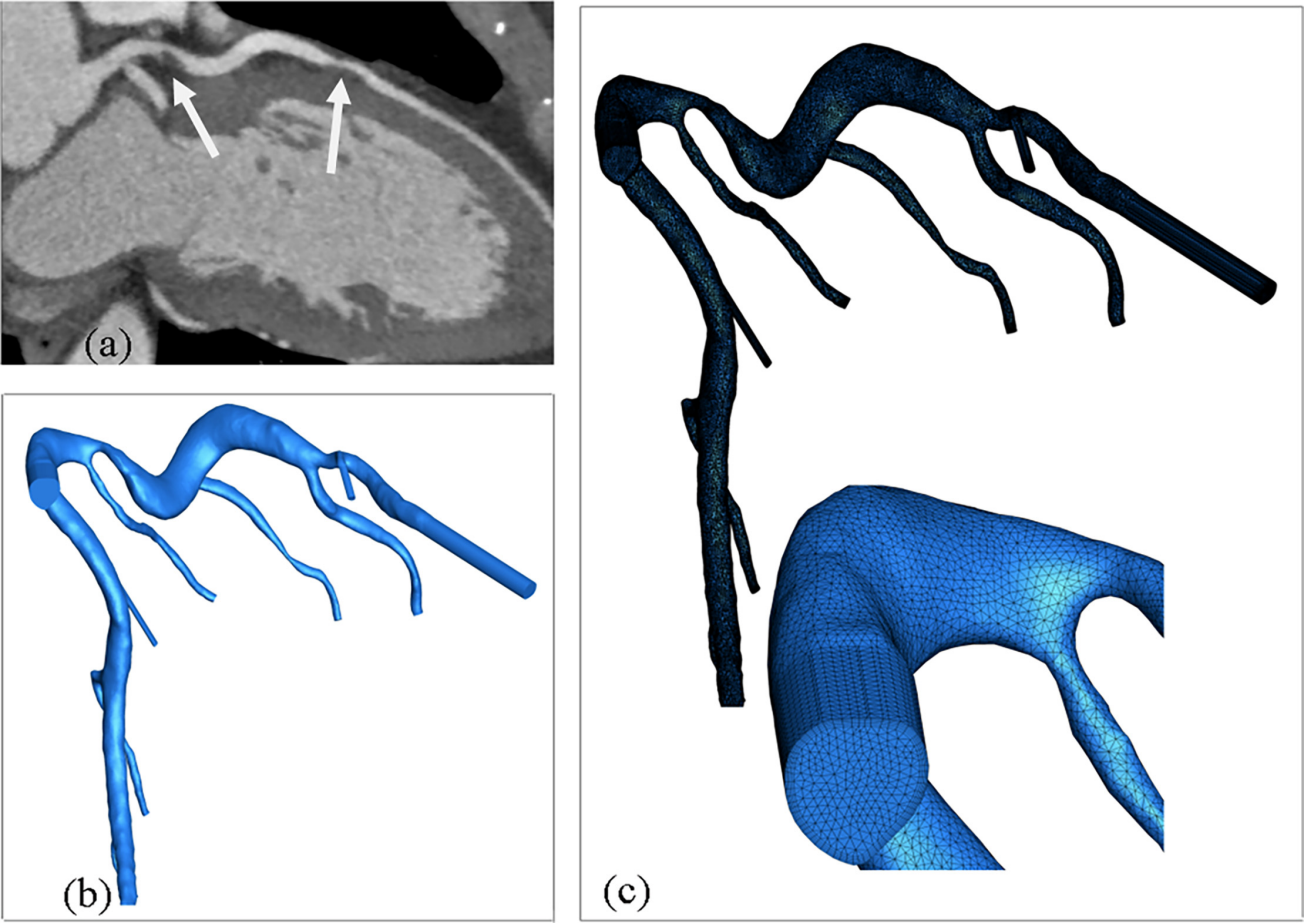
(a) Coronary CTA images for a 52-years-old man, which showed two stenoses (indicated by white arrows) along the left anterior descending artery (b) 3D coronary model reconstructed from CTA images and (c) meshes generated for this model with enlarged view of dense meshes assigned near the wall.

A 3D coronary artery tree was reconstructed from CTA images through image processing. An example of CTA images is shown in Fig 1A where the stenoses were observed along the left anterior descending artery (LAD). The reconstructed model showed that the coronary lumens were far from circular in the stenotic regions. Meshes were generated (Fig 1C) for the 3D model to facilitate CFD iterations. In this way, CTA-derived non-invasive FFR_SS_ can be calculated.

**Fig 2:**
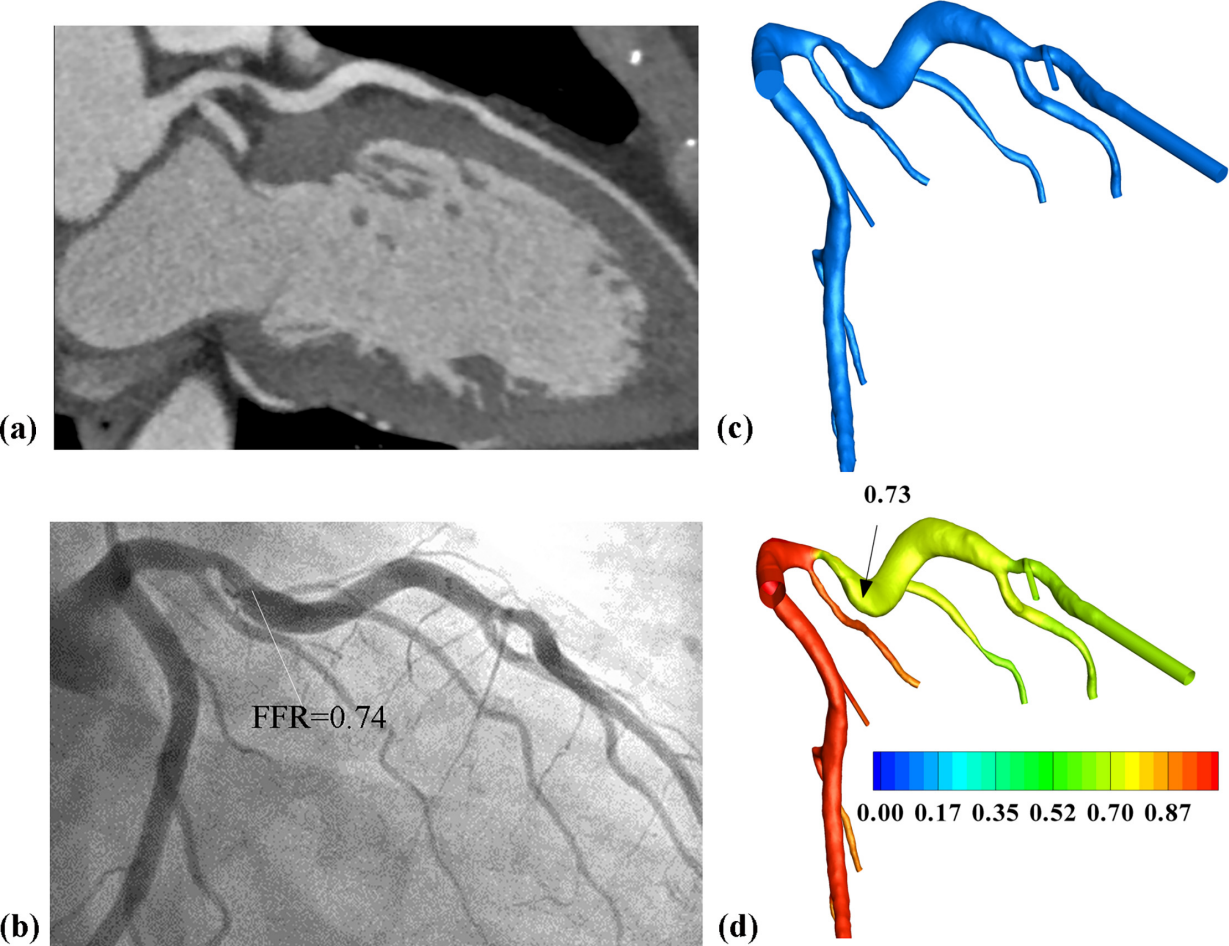
Representative patient with functional significant stenosis. Severe luminal stenosis was observed from (a) CTA images and (b) invasive angiography measured FFR at 0.74 (FFR≤0.8). Based on the (c) 3D model reconstructed from CTA images, CFD derived (d) pressure distributions on the 3D model and the calculated FFR_SS_ was 0.73.

**Fig 3:**
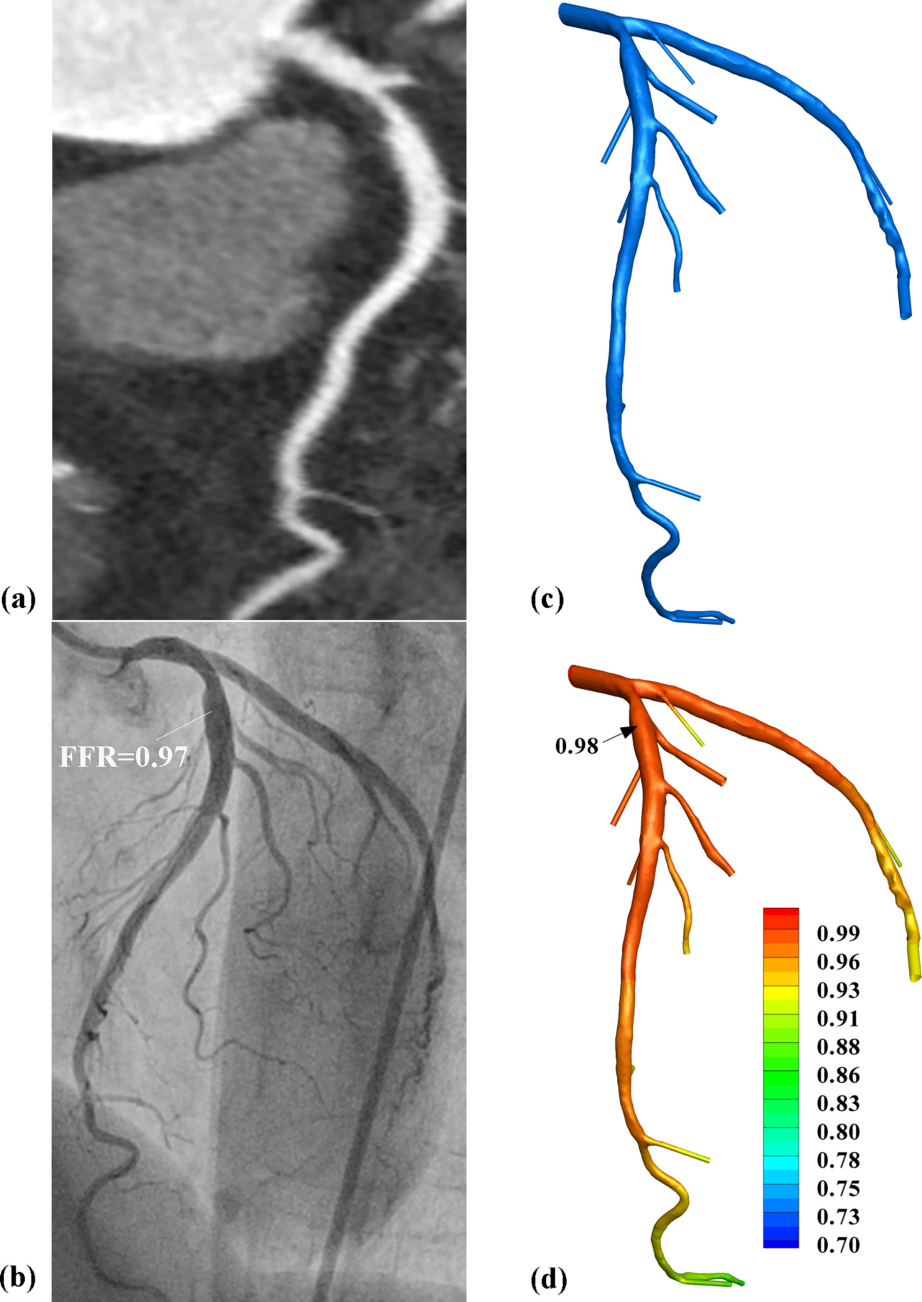
Representative patient with functional non-significant stenosis. Moderate luminal stenosis was observed from (a) CTA images and (b) invasive angiography measured FFR at 0.97 (FFR>0.8). Based on the (c) 3D model reconstructed from CTA images, CFD derived (d) pressure distributions on the 3D model and the calculated FFR_SS_ was 0.98.

Two representative examples are shown in Figs 2 and 3 separately. Based on CTA, severe (Fig 2A) and moderate (Fig 3A) stenoses were observed for two patients, respectively. One stenosis turned out to be functionally significant (Fig 2B) while the other non-significant (Fig 3B) with measured FFR of 0.74 and 0.97, respectively. Based on the CTA-derived CFD, the pressure distributions were calculated on these two models and the calculated FFR_SS_ were 0.73 and 0.98, respectively. Using the analytical model, the calculated FFR_AM_ were 0.67 and 0.97 separately for these two stenoses.

**Fig 4:**
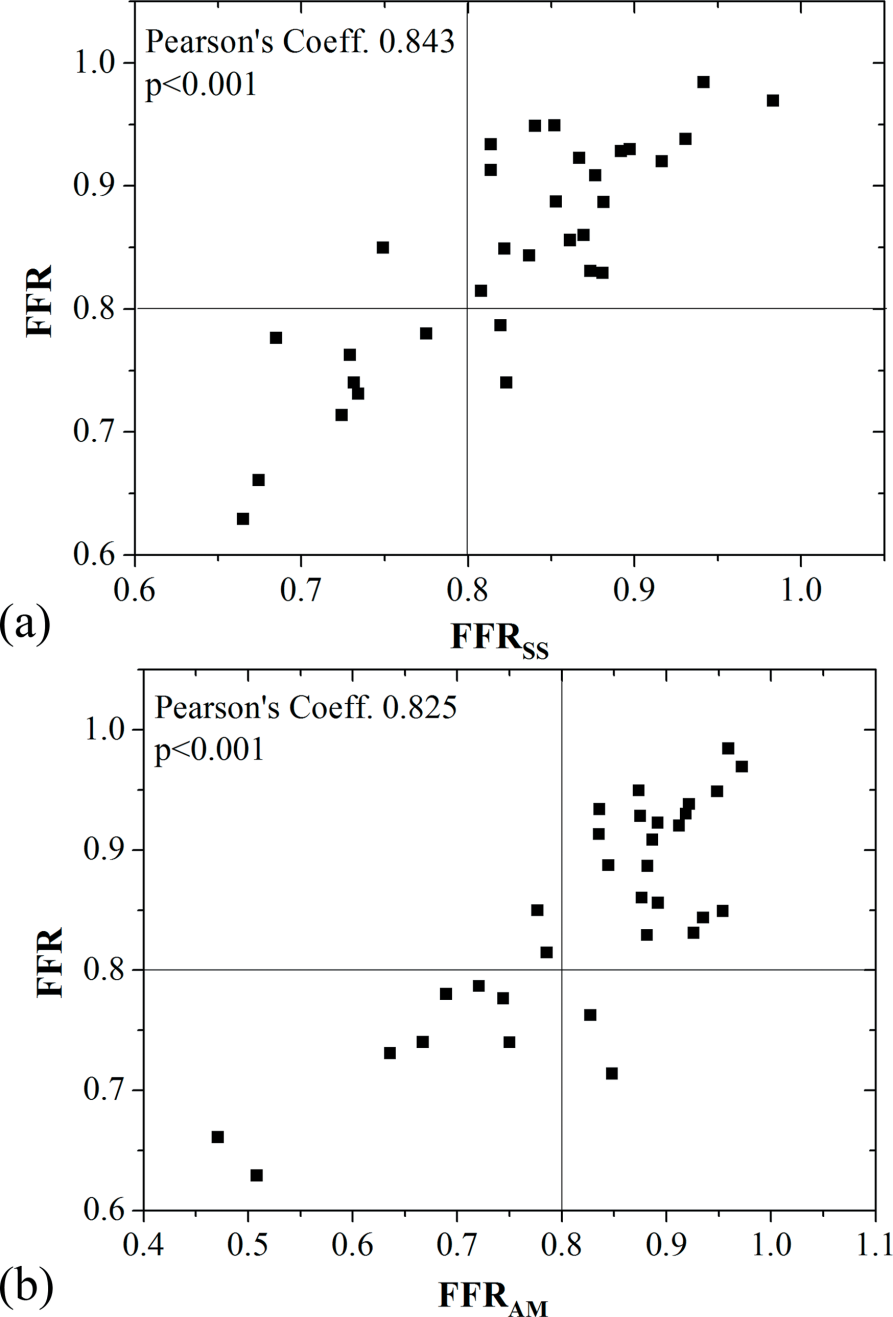
Correlation between FFR with (a) FFR_SS_ and (b) FFR_AM_ for a total of 32 stenoses.

**Fig 5:**
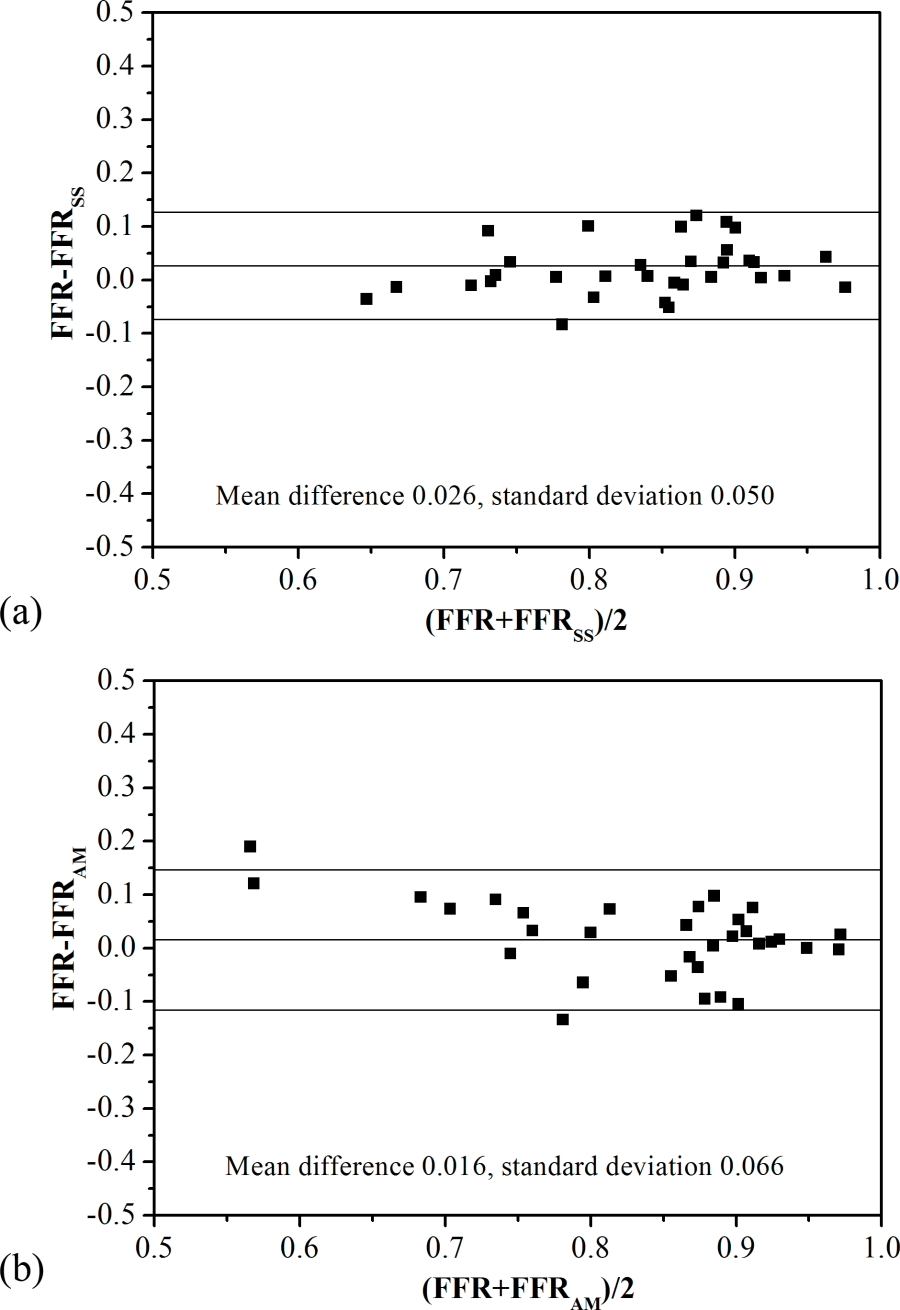
Bland-Altman plot of (a) FFR with FFR_SS_ and (b) FFR with FFR_AM_ on a per-vessel basis.

Table 2 tabulates both hemodynamic indices obtained through invasive angiography and those derived non-invasively from CTA. Fig 4A and 4B show the correlation of FFR_SS_ and FFR_AM_ with FFR, respectively. Both FFR_SS_ (Pearson’s correlation coefficient 0.843, p<0.001) and FFR_AM_ (Pearson’s correlation coefficient 0.825, p<0.001) showed strong correlation with FFR. Both FFR_SS_ (mean difference 0.026, standard deviation 0.050, Fig 5A) and FFR_AM_ (mean difference 0.016, standard deviation 0.066, Fig 5B) demonstrated slight but non-significant differences as compared with FFR in assessing the functional significance of stenosis on a per-vessel basis.

### Diagnostic Performance of AS, DS, FFR_SS_ and FFR_AM_ for Diagnosis of Ischemia

**Fig 6:**
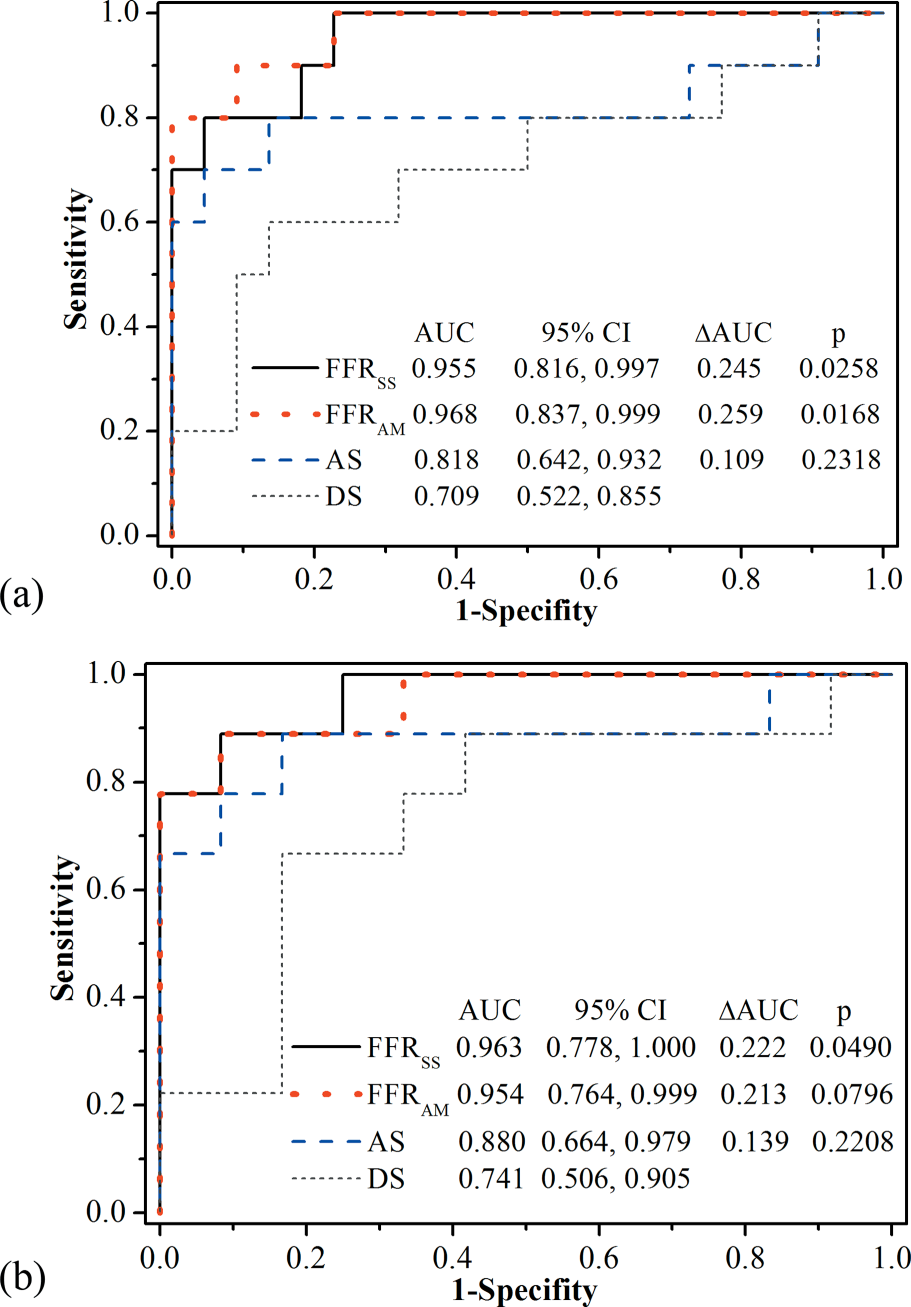
Area under the receiver-operating characteristic curve (AUC) of FFR_SS_, FFR_AM_, AS and DS and the difference between AUCs of FFR_SS_ (FFR_AM_ and AS) and DS for discriminating ischemic stenosis on a (a) per-vessel and (b) per-patient basis separately.

In contrast to the gold standard of FFR, CTA-derived hemodynamic indices of FFR_SS_ (AUC was 0.955 and 0.963 on a per-vessel and per-patient basis, respectively) and FFR_AM_ (AUC was 0.968 and 0.954 on a per-vessel and per-patient basis, respectively) have higher AUC than AS (AUC was 0.818 and 0.880 on a per-vessel and per-patient basis respectively) and DS (AUC was 0.709 and 0.741 on a per-vessel and per-patient basis respectively) measured from CTA on both per-vessel (Fig 6A) and per-patient (Fig 6B) bases. The difference between AUCs of FFRss and DS was significant (p<0.05) on both per-vessel and per-patient bases.

By combining CTA with steady state flow simulation and analytical models separately, the diagnosis accuracy of both FFR_SS_ (90.6%) and FFR_AM_ (87.5%) was higher than that of anatomical index of either AS (75.0%) or DS (75.0%) from CTA alone, on a per-vessel basis (Table 3). Higher specificity was found for CTA-derived FFR_SS_ (95.5%) than AS (72.7%) and DS (86.4%) derived from CTA alone, with the same (in contrast to AS) or improved (in contrast to DS) sensitivity, which were 80.0%, 80.0% and 50.0% respectively on a per-vessel basis. CTA-derived hemodynamic index (either FFR_SS_ or FFR_AM_) was found to enhance both PPV and NPV than CTA-based anatomic index (either DS or AS) on a per-vessel basis. The enhancement of diagnostic performance by replacing anatomic index of either DS or AS to CTA-derived hemodynamic index (either FFR_SS_ or FFR_AM_) was also found on a per-patient basis (Table 3).

**Table 3:** Diagnostic performance of FFR_SS_, FFR_AM_, AS and DS compared to invasively measured FFR for discrimination of ischemic coronary stenosis (ischemic group, FFR≤0.8; non-ischemic group, FFR>0.8)

	Criterion to discriminate ischemic stenosis	Accuracy (%)	Sensitivity (%)	Specificity (%)	PPV (%)	NPV (%)
**Per-vessel**	FFR_SS_ ≤0.8 (95% CI)	90.6 (75.0–98.0)	80.0(44.4–97.5)	95.5 (77.2–99.9)	88.9 (51.8–99.7)	91.3 (72.0–98.9)
	FFR_AM_≤0.8 (95% CI)	87.5 (71.0–96.5)	80.0 (44.4–97.5)	90.9 (70.8–98.9)	80.0 (44.4–97.5)	90.9 (70.8–98.9)
	AS≥62% (95% CI)	75.0 (56.6–88.5)	80.0 (44.4–97.5)	72.7 (49.8–89.3)	57.1 (28.9–82.3)	88.9 (65.3–98.6)
	DS≥50% (95% CI)	75.0 (56.6–88.5)	50.0 (18.7–81.3)	86.4 (65.1–97.1)	62.5 (24.5–91.5)	79.2 (57.9–92.9)
**Per-patient**	FFR_SS_ ≤0.8 (95% CI)	90.5 (69.6–98.8)	88.9 (51.8–99.7)	91.7 (61.5–99.8)	88.9 (51.8–99.7)	91.7 (61.5–99.8)
	FFR_AM_≤0.8 (95% CI)	85.7 (63.7–97.0)	77.8 (40.0–97.2)	91.7 (61.5–99.8)	87.5 (47.4–99.7)	84.6 (54.6–98.1)
	AS≥62% (95% CI)	76.2 (52.8–91.8)	80.0 (44.4–97.5)	72.7 (39.0–94.0)	72.7 (39.0–94.0)	80.0 (44.4–97.5)
	DS≥50% (95% CI)	71.4 (47.8–88.7)	55.6 (21.2–86.3)	83.3 (51.6–97.9)	71.4 (29.0–96.3)	71.4 (41.9–91.6)

PPV: Positive Predictive Value; NPV: Negative Predictive Value; CI: Confidence Interval.

### Effect of Individual Hyperemic Response on the Calculated FFR_SS_

**Fig 7:**
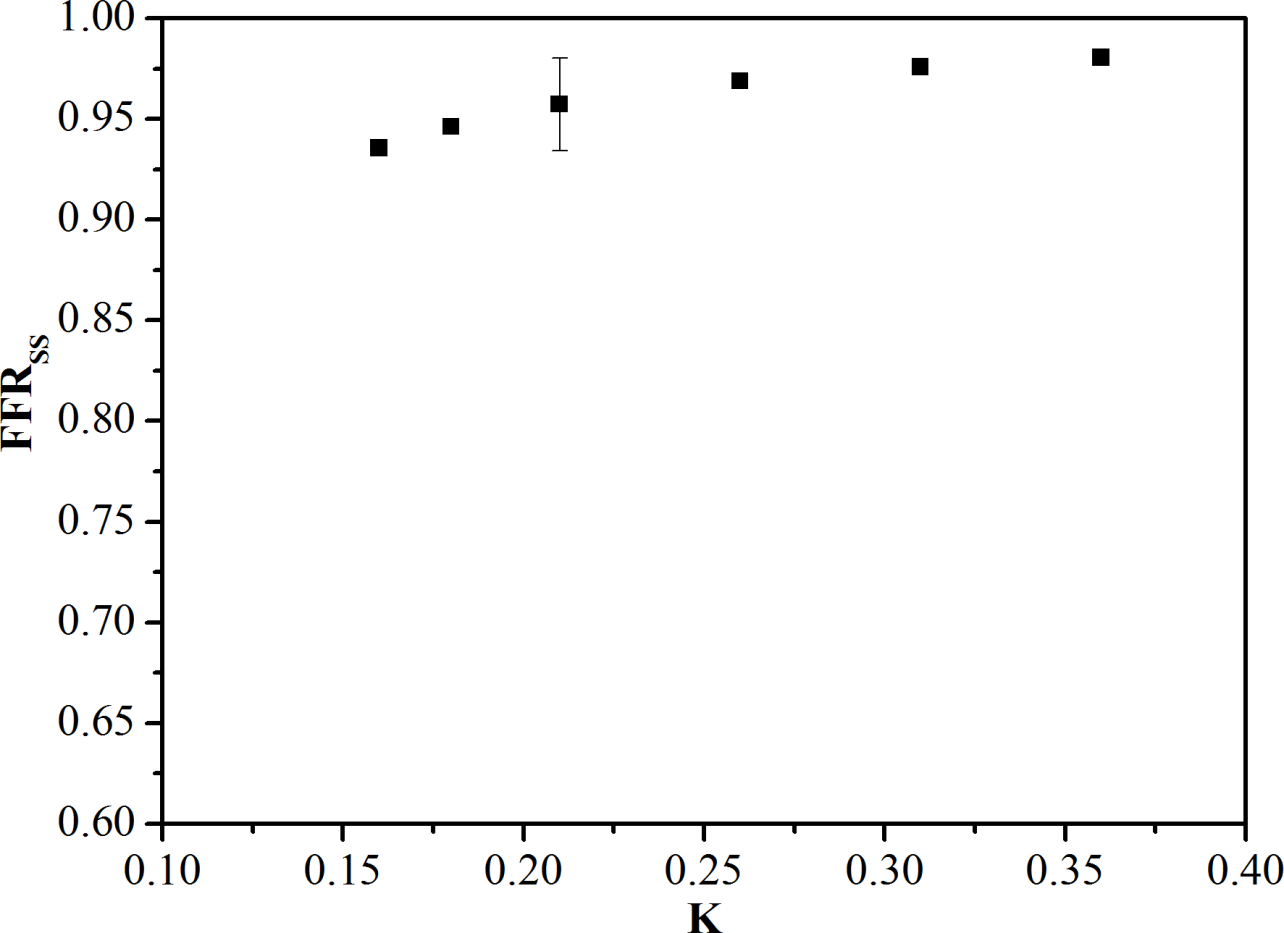
Sensitivity of FFR_SS_ with a varied K.

To investigate the effects of individual hyperemic response on the calculated FFR_SS_, we carried out a series of numerical simulations on a single patient-specific model and varied the values of K. It was found that FFR_SS_ increased with increasing K (Fig 7). With K ranging from 0.16 to 0.36 (within physiological hyperemia response range [35]), the maximum variation of FFR_SS_ was less than 2.4%, compared to the FFR_SS_ value obtained at K = 0.21.

## Discussion

### Non-invasive Assessment of Anatomic Significance of Stenosis using CTA

As a non-invasive examination, CTA is used to characterize the anatomic severity (DS and AS) of CAD with fair medical cost and less complications than invasive procedures. In this study, the anatomic parameters of DS and AS were measured for each stenosis of patient-specific model reconstructed from CTA images. The inter-operator variability of DS (5.6%) was higher than AS (1.5%). This is because the determination of DS is somewhat subjective, owing to the difficulty of delineating both stenotic and normal reference segments under multiple pathological states (e.g., diffuse CAD, high-flow coronary fistulae and ectasia). In contrast to DS, AS had less dependency of projection plane and reference sites [30].

As anatomical measures, DS and AS measured from CTA cannot characterize the hemodynamic and functional significance of CAD on myocardial blood supply. In this study, 8 vessels were found to have DS ≥ 50%, while only 5 vessels led to FFR ≤ 0.8. This is consistent with the findings that using the anatomic index of DS to diagnose ischemia in stenosis leads to a much higher false positive rate relative to FFR [37]. These results appear to confirm the clinical findings [5,37] that DS tends to overestimate the significance of stenosis for ischemia [38,39].

In contrast to DS (AUC was 0.709 and 0.741 on a per-vessel and per-patient basis separately), AS better (but not significant, p>0.05) discriminated non-ischemic and ischemic groups (AUC was 0.818 and 0.880 on a per-vessel and per-patient basis separately). This was because the pressure drop over a stenotic region was largely attributed to convective and diffusive energy losses due to constriction and sudden expansion of the lumen area distal to the stenosis [23]. The accuracy of AS was limited (75.0% on a per-vessel basis), however, due to the difficulty to delineate both the stenotic and the normal reference segments under many pathological states (such as diffuse CAD, high-flow coronary fistulae, ectasia, etc.), the limitations of imaging resolution, the effects of stenosis location and length of the stenosis, exit angle, and other physiological factors on the hemodynamics of CAD [40].

### Non-invasively Assessment of Hemodynamic Significance of Stenosis via FFR_SS_

In this study, FFR_SS_ was obtained non-invasively by combining steady state flow simulation with CTA images. The novel specification of boundary conditions greatly reduced the computational time as compared to those of pulsatile flow simulations [22]. The pulsatile flow simulations were used to compute FFR_CT_ for the trials of DISCOVER-FLOW [16], DeFACTO [18] and NXT [19] and it took 6 hours [16] or 1–4 hours [19] for CFD analysis per examination. Direct comparison of the computational time between FFR_CT_ trials and current study is not practical, as the hardware specifications of the workstations [16,18,19] was not known. Our previous study showed that the computational time with steady flow simulation was only 1/16 time of that in pulsatile flow simulation [22], using the same workstation, which demonstrated the significant reduction of computational time with our proposed approach.

In this pilot study, FFR_SS_ was found to have very high diagnostic performance in contrast to invasively measured FFR. The correlation between FFR_SS_ and FFR in this study (Pearson’s correlation coefficient 0.843, p<0.001) was equivalent with that of NXT (Pearson’s correlation coefficient 0.82, p<0.001), DeFACTO (Pearson’s correlation coefficient 0.63) and Discover-Flow (Pearson’s correlation coefficient 0.72) trials. Similar level of the accuracy and AUC (on a per-patient basis) in this study (90.6% and 0.955 respectively) was observed in contrast to those of NXT (81% and 0.90 respectively), DeFACTO (73% and 0.81 respectively) and Discover-Flow (87.4% and 0.92 respectively) trials.

The slight improvement of correlation, accuracy and AUC in this study may be due to the two reasons. First, it may be attributed to the improvement of CT image quality. In this study, the majority of the patients (n = 20, 95%) received CTA examination with a 320 slice Toshiba Aquilion ONE^TM^ CT scanner and 1 patient with 64 slice multi-detector CTA scanner (Toshiba Aquilion 64) in the same center. CTA was performed using CTA scanners, however, with a minimum of 64 detector rows in multiple centers for NXT trial [19]. The CTA scanner information was not provided in [19], but the scanners were listed as LightSpeed VCT/Discovery from GE, SOMATOM Sensation and Definition from Siemens, Brilliance 256 and 64 from Philips, Aquilion ONE and 64 from Toshiba in [41] for DeFACTO trial. Because Toshiba Aquilion CT scanners have finer detector elements of 0.5 mm than many other CT scanners (GE LightSpeed VCT: 0.625mm; Siemens Sensation 64: 0.6mm; Philips Brilliance 64: 0.625mm), the scanner used in this pilot study might have higher image resolution than some of the centers participating in the NXT trial and the image quality may have been better and more consistent. This finding was in agreement with NXT trial, which reported that the enhancement of diagnostic performance of FFR_CT_ was related to the increased CT image quality, comparing with the trials of DISCOVER-FLOW [16] and DeFACTO [18]. Second, it may be due to the consistent CTA protocol and imaging processing techniques applied in our center, which minimize the propagation of anatomic structure variations into the errors of FFR_SS._

A recent study attempted to reduce the computational time to 5–10 minute per patient but found a moderate (Pearson’s correlation coefficient 0.59) correlation between their calculated FFR and invasive FFR [21]. Although the poor correlation may be due, in part, to higher Agatston coronary calcium score (555±542) in their study than our study (333 ±370), their method to simplify the flow in 3D vessel as 1-D flow may lead to oversimplification of coronary blood flow mechanics and results in significant error in the CTA-derived FFR calculation.

### Non-invasive Analytical Assessment of Hemodynamic Significance of Stenosis via FFR_AM_

In this pilot study, the hemodynamic index of FFR_AM_ was found to have good diagnostic performance when compared with the gold standard of FFR. The correlation between FFR_AM_ and FFR was high (Pearson’s correlation coefficient 0.825, p<0.001). Based on Bland-Altman plot (Fig 5B), there was no significant difference between FFR_AM_ and FFR (mean difference 0.016). In contrast to FFR_CT_ (Pearson’s correlation coefficient was 0.63 to 0.82 [16,18,19]), FFR_AM_ had closer correlation with FFR (0.83 separately). Equivalent accuracy of FFR_AM_ in this study (87.5% for per-vessel analysis) was also observed in contrast to those of FFR_CT_ studies (84.3% to 86% for per-vessel analysis) [16,18,19]. Therefore, the analytical model [23] has potential to predict myocardial FFR. Although the diagnostic performance of FFR_AM_ (AUC = 0.95) was slightly worse than FFR_SS_ (AUC = 0.96) for per-patient analysis, the real time calculation (negligible computational time) is an advantage.

The discrepancy between FFR_AM_ and FFR_SS_ may be due to the simplification of the analytical model. The former was largely dependent on the measurement of stenosis dimensions (proximal, distal and minimal lumen area, and length) and hyperemic coronary flow rate through the lesion. It is worth noting that the hyperemic coronary flow rate used in this study was from the numerical simulation. Since coronary angiography can quantify coronary blood flow [42,43], the analytical formulation has utility in angiography or in future developments of fast CTA, echocardiography or magnetic resonance angiography that may provide simultaneous coronary images and flow rate.

### Limitations of the Study

This study has several limitations. First, the computational models may not capture the tremendous biological variability between CAD patients. For example, FFR is measured by pressure wire at the hyperemia state. Wilson et al. [35] reported that the total coronary resistance was reduced from 0.16 to 0.36 (*K*) times its resting value following an intracoronary adenosine bolus of 16μg (intracoronary adenosine infusion at 120–240 μg/min or intravenous adenosine infusion at 140 μg/kg/min). We found that FFR_SS_ increased with increasing *K*. This was because higher downstream vascular resistance led to a reduction in the flow and pressure drop over the stenosis, resulting in higher FFR_SS_. Within physiological hyperemia response range [35] (*K* ranging from 0.16 to 0.36), the maximum variation of FFR_SS_ was less than 2.4%, compared to the FFR_SS_ value obtained at *K* = 0.21. Therefore, FFR_SS_ was not particularly sensitive to the individual hyperemic response in patients free of microvascular disease. The assumption of normal microvascular function is one of the limitations of this study. Our recruited patients have no prior myocardial infarction (MI) or PCI in the vessel territories interrogated with invasive FFR measurement, however, which implies that their microvascular dysfunction is minimal. Furthermore, only 2 patients presented diabetes. The assumption of normal microvascular function was also applied to other non-invasive FFR studies [16,17,20]. The diagnostic performance of FFR_SS_ can be further examined in a myriad of patient cohorts [44], including those patients with left ventricular hypertrophy, diabetic (with increased fibrosis), prior MI or PCI, who may display important differences in microvascular resistance.

Second, the simulations assumed the coronary artery vessels as rigid and did not consider the motion of compliant vessel wall and the heart during the cardiac cycle. The movement of the heart and compliant vessel walls was reported to be less important, however, in coronary arteries than the geometry and boundary conditions on the pressure and flow fields [45,46].

Third, only time-averaged flow patterns and pressure distributions were predicted by the steady state flow simulation in this study. Although the deduced FFR_SS_ was comparable to FFR measured with pressure wire, detailed pulsatile flow simulation as in [20] will be necessary if transient pressure waveforms are of interest.

Fourth, although the addition of FFR_SS_ to coronary CTA is possible, adoption of FFR_SS_ in clinical domain remains challenging in view of the following: 1) inherently lower diagnostic accuracy of intermediate stenosis, when FFR is close to the cut point, even for the repeated invasive measurement of FFR [47] and 2) necessary introduction of a number of physiological assumptions for CFD. Therefore, additional investigations on larger appropriately designed prospective trials are necessary to provide more definitive data.

Finally, this is a single center study. The sample size was relatively small and the patients with previous coronary intervention were excluded. Further study on a larger population with wider recruitment criteria is necessary to verify and expand the present findings.

### Significance

Although non-invasively derived FFR from CTA images has been attempted by several groups, it may lead to long computational times [16,18,19] or moderate accuracy [21]. A major contribution of our study is a steady flow simulation algorithm that can reduce the computational burden [13] to 1/16 compared with pulsatile flow simulation [22] but maintains high accuracy in relation to FFR (Pearson’s correlation coefficient 0.843 (p<0.001) for FFR_SS_ and 0.82 (p<0.001) for FFR_CT_ [19]). An additional contribution is the clinical validation of an analytical model for deriving FFR_AM_ in real time. These simplified models that maintain accuracy may add to the current developments of non-invasive measurements of FFR for diagnosis of intermediate CAD lesions.

## Supporting Information

S1 Appendix (DOCX)

S2 Appendix (DOCX)
