# Supplementary material for: Simplified Models of Non-Invasive Fractional Flow Reserve Based on CT Images
Source: PLoS One. 2016 May 17;11(5):e0153070. doi: 10.1371/journal.pone.0153070 (PMC4871505; doi:10.1371/journal.pone.0153070)
Supplement: S1 Appendix — (DOCX) [file pone.0153070.s001.docx]

# S1 Appendix

To simulate blood flow in the patient-specific coronary artery tree models, this study used FLUENT^TM^ to solve continuity and Navier-Stokes equations as shown in Eqs. A1 and A2 respectively.

$\frac{\partial u_{j}}{\partial x_{j}}=0$ (A1)

$\rho\frac{\partial\left(u_{j}u_{i}\right)}{\partial x_{j}}=-\frac{\partial P}{\partial x_{i}}+\mu\frac{\partial}{\partial x_{j}}\left(\frac{\partial u_{i}}{\partial x_{j}}+\frac{\partial u_{j}}{\partial x_{i}}\right)$ (A2)

where *x_j_* is the location in Cartesian coordinates, *u_j_* (or *u_i_*) is the Cartesian component of velocity, *P* represents the static pressure; and *ρ* and *μ* were set as 1,060 kg/m^3^ and 4.5×10^-3^ Pa·s respectively to represent the density and dynamic viscosity of blood in large epicardial arteries [1,2].

Pressure and resistance boundary conditions were specified at the inlet and outlets of each model respectively to mimic physiological conditions. To assign the inlet total pressure, patient-specific systolic and diastolic brachial pressure was used to calculate the mean brachial pressure and match the mean aortic pressure.

A key element in assigning coronary resistance values of the outlets is to prescribe reference pressure (P_0_). Physiologic studies report that coronary pressure flow lines are concave to the axis of flow at lower pressures but straight at physiological pressures [3,4]. The zero flow pressure intercept at the physiologic pressure range (i.e., P_0_ in this study) exceeds coronary venous or left ventricular diastolic pressure by five to ten-fold [5]. Therefore, we propose a novel method in this study to determine the reference pressure P_0_, and therefore *R_i_* through an iterative procedure. The details are provided as following.

Briefly, the total resistance $R_{\mathrm{total}}$*R_inlet_* at resting condition is defined by Eq. A3

$R_{\mathrm{total}}=(P-P_{0})/Q_{\mathrm{total}}$ (A3)

where *P_inlet_* represents the mean aortic pressure, estimated from the mean branchial artery pressure. The total coronary flow at resting can be estimated from the myocardial mass [6,7]. Accordingly *Q_inlet_* can be determined from CT images.

At each coronary outlet$,$

$P_{i}=P_{0}+R_{i}Q_{i}$ (A4)

The correlation between the resistance of downstream vasculature of each coronary branch (*R_i_*) and that of the coronary tree (*R_inlet_*) can be estimated from the scaling law [8],

$R_{i}=N_{i}R_{\mathrm{inlet}}$ (A5)

for non-stenotic vessels. Because the resistance of downstream vasculature was determined by the inherent anatomy rather than by upstream structures [9], the resistance of vessels with stenosis satisfied the same relationship as Eq. A5.

From A3 and A5, *R_i_* can have the following form:

$R_{i}=N_{i}\frac{P_{\mathrm{inlet}}-P_{0}}{Q_{\mathrm{inlet}}}=\frac{P_{i}-P_{0}}{Q_{i}}$ (A6)

By the mass conservation law, total outflow was equal to inflow (Eq. A7),

$\sum_{i=1}^{N} Q_{i}=Q_{\mathrm{inlet}}$ (A7)

From Eqs. A4, A5 and A7, it can be shown that *P_0_* is given by:

$P_{0}=P_{\mathrm{inlet}}-\sum_{i=1}^{N} \left( \frac{P_{i}-P_{0}}{N_{i}} \right)$ (A8)

$R_{i}=N_{i}\frac{P_{\mathrm{inlet}}-P_{0}}{Q_{\mathrm{inlet}}}$ At hyperemia, coronary microcirculation was assumed to have predictable response to adenosine and the resistance was assumed to be reduced to *K* times the resting values (where *K*=0.21), a value within the physiological range measured by Wilson et al. [10]. From Eq. (A6), the hyperemic resistance is given by:

$R_{i,hypermia}=KR_{i}=KN_{i}\frac{P_{\mathrm{inlet}}-P_{0}}{Q_{\mathrm{inlet}}}$ (A9)

In order to ensure smooth convergence during the numerical iterations of CFD, *P_0_* and *R_i_* were initialized as 20 mmHg and 1.0e+8 Pa·s/m^3^, respectively; and then updated with a under-relaxation scheme as formulated in Eqs. A10 and A11 until the total outflow from all the outlets matched the inflow rate at hyperemia.

$P_{0, new}={\left( 1-\alpha\right)P_{0,old}+\alpha(P}_{\mathrm{inlet}}-\sum_{i=1}^{N} \left( \frac{P_{i}-P_{0}}{N_{i}} \right))$ (A10)

$R_{i,hypermia,new}=\left( 1-\alpha\right)R_{i,hypermia,old}+\alpha(KN_{i}\frac{P_{\mathrm{inlet}}-P_{0}}{Q_{\mathrm{inlet}}})$ (A11)

Here, *α* was under-relaxation factor. P_0_*_,old_* and *R_i,hyperemia, old_* represented the reference pressure and resistance in the last iteration, while *P_0,new_* and *R_i,hyperemia,new_* represented their values in the next iteration. As minimal microvascular resistance was found to be independent of epicardial stenosis severity [11], hyperemic microcirculatory resistance distal to a stenosis was assumed to be the same as that for coronary arteries free of stenosis [12] as specified in Eq. A11.

With these novel iterative boundary conditions, the computational burden can be reduced in contrast to solving the complex lumped-parameter heart and coronary models described in Ref. 16. The calculated value of P_0_ was 38.6 ± 9.5 mmHg for the 21 patients investigated in this study, which was close to 37.9 ± 9.8 mmHg measured by Dole et al. [5] for 10 patients and 36±9 mmHg measured by Nanto et al. [13] for 15 subjects respectively at vasodilated state. A no-slip boundary condition was imposed on the vessel wall.

**References**

1. Huo Y, Wischgoll T, Kassab GS. Flow patterns in three-dimensional porcine epicardial coronary arterial tree. Am J Physiol Heart Circ Physiol. 2007;293: H2959–H2970. doi:10.1152/ajpheart.00586.2007

2. Zhang JM, Chua LP, Ghista DN, Yu SCM, Tan YS. Numerical investigation and identification of susceptible sites of atherosclerotic lesion formation in a complete coronary artery bypass model. Med Biol Eng Comput. 2008;46: 689–699. doi:10.1007/s11517-008-0320-4

3. Klocke FJ, Weinstein IR, Klocke JF, Ellis AK, Kraus DR, Mates RE, et al. Zero-flow pressures and pressure-flow relationships during single long diastoles in the canine coronary bed before and during maximum vasodilation. Limited influence of capacitive effects. J Clin Invest. 1981;68: 970–980. doi:10.1172/JCI110351

4. Spaan JAE, Piek JJ, Hoffman JIE, Siebes M. Physiological basis of clinically used coronary hemodynamic indices. Circulation. 2006. pp. 446–455. doi:10.1161/CIRCULATIONAHA.105.587196

5. Dole WP, Richards KL, Hartley CJ, Alexander GM, Campbell AB, Bishop VS. Diastolic coronary artery pressure-flow velocity relationships in conscious man. Cardiovasc Res. 1984;18(9): 548–554.

6. Hamada M, Kuwahara T, Shigematsu Y, Kodama K, Hara Y, Hashida H, et al. Relation between coronary blood flow and left ventricular mass in hypertension: noninvasive quantification of coronary blood flow by thallium-201 myocardial scintigraphy. Hypertens Res. 1998;21: 227–234.

7. Wieneke H, von Birgelen C, Haude M, Eggebrecht H, Möhlenkamp S, Schmermund A, et al. Determinants of coronary blood flow in humans: quantification by intracoronary Doppler and ultrasound. Journal of Applied Physiology. 2005. doi:10.1152/japplphysiol.00724.2004

8. Zhou Y, Kassab GS, Molloi S. On the design of the coronary arterial tree: a generalization of Murray’s law. Phys Med Biol. 1999;44: 2929–2945. doi:10.1088/0031-9155/44/12/306

9. Kim H, Vignon-Clementel I, Coogan J, Figueroa C, Jansen K, Taylor C. Patient-specific modeling of blood flow and pressure in human coronary arteries. Ann Biomed Eng. Springer Netherlands; 2010;38: 3195–3209. doi:10.1007/s10439-010-0083-6

10. Wilson RF, Wyche K, Christensen BV, Zimmer S LD. Effects of adenosine on human coronary arterial circulation. Circulation. 1990;82: 1595–1606. doi:10.1161/01.CIR.82.5.1595

11. Aarnoudse W, Fearon WF, Manoharan G, Geven M, Van De Vosse F, Rutten M, et al. Epicardial stenosis severity does not affect minimal microcirculatory resistance. Circulation. 2004;110: 2137–2142. doi:10.1161/01.CIR.0000143893.18451.0E

12. Taylor CA, Fonte TA, Min JK, City R, Angeles L. Computational fluid dynamics applied to cardiac computed tomography for noninvasive quantification of fractional flow reserve: scientific basis. J Am Coll Cardiol. 2013;61: 2233–2241. doi:10.1016/j.jacc.2012.11.083

13. Nanto S, Masuyama T, Takano Y. Determination of coronary zero flow pressure by analysis of the baseline pressure – flow relationship in humans. Jpn Circ J. 2001;65 (9): 793–796.
