# Supplementary material for: Simplified Models of Non-Invasive Fractional Flow Reserve Based on CT Images
Source: PLoS One. 2016 May 17;11(5):e0153070. doi: 10.1371/journal.pone.0153070 (PMC4871505; doi:10.1371/journal.pone.0153070)
Supplement: S2 Appendix — (DOCX) [file pone.0153070.s002.docx]

# S2 Appendix

We used an analytical model [23] to predict the pressure drop over a stenosis (∆P_1_). As longer stenosis led to larger pressure drop due to entrance effect, a dimensionless radius of inviscid core (γ) was first solved with Eq. B1 to determine whether to include the entrance effect as:

$\frac{\pi\mu L}{4\rho Q}=\frac{1}{4}\int_{\gamma}^{1} \frac{(1-\gamma)(6+\gamma)(1+4\gamma+9\gamma^{2}+4\gamma^{3})}{5\gamma\left(3+2\gamma\right)\left(3+2\gamma+\gamma^{2}\right)^{2}}d\gamma$ (B1)

*L* and *Q* represented the stenosis length and flow rate, respectively. *μ* and *ρ* were the viscosity and density of the blood.

If γ≥0.05, the stenosis length was short and the entrance effect can be ignored. Therefore, the pressure drop in a vessel with a single stenosis can be calculated as follows:

$\begin{matrix} \Delta P_{1}=\frac{\rho Q^{2}}{2}\left(\frac{1}{A_{distal}^{2}}-\frac{1}{A_{proximal}^{2}}\right)+\frac{\rho Q^{2}}{2A_{stenosis}^{2}}\frac{96}{5}\times\int_{\gamma}^{1} \frac{(1+4\gamma+9\gamma^{2}+4\gamma^{3})}{\gamma(3+2\gamma)\left(3+2\gamma+\gamma^{2}\right)^{2}}d\gamma+\frac{\rho Q^{2}}{2}\left\{\left(\frac{1}{A_{stenosis}}-\frac{1}{A_{distal}}\right)^{2}\right. \\ \begin{matrix} & \end{matrix}\left.+\left.\left[2\left(\frac{1}{A_{stenosis}}-\frac{1}{A_{distal}}\right)\left(\frac{1}{A_{stenosis}}-\frac{1}{3A_{distal}}\right)-\left(\frac{1}{A_{stenosis}}-\frac{1}{A_{distal}}\right)^{2}\right.\right]\left(1-\gamma\right)^{2}\right\} \end{matrix}$ (B2)

*A_distal_*, *A_proximal_* and *A_stenosis_* represented the lumen area at the distal, proximal and stenotic locations, respectively.

For long stenoses (γ<0.05), the entrance effect was considered and the total pressure drop over a single stenosis was calculated as:

$\begin{matrix} \Delta P_{1}=\frac{\rho Q^{2}}{2}\left(\frac{1}{A_{distal}^{2}}-\frac{1}{A_{proximal}^{2}}\right)+\frac{\rho Q^{2}}{2A_{stenosis}^{2}}\frac{96}{5}\times\int_{0.05}^{1} \frac{(1+4\gamma+9\gamma^{2}+4\gamma^{3})}{\gamma(3+2\gamma)\left(3+2\gamma+\gamma^{2}\right)^{2}}d\gamma\\ \begin{matrix} & \end{matrix}+\rho Q^{2}\left(\frac{1}{A_{stenosis}}-\frac{1}{A_{distal}}\right)\times\left(\frac{1}{A_{stenosis}}-\frac{1}{3A_{distal}}\right) \end{matrix}$ (B3)
